# Applying Sensor-Based Information Systems to Identify Unplanned Downtime in Mining Machinery Operation

**DOI:** 10.3390/s22062127

**Published:** 2022-03-09

**Authors:** Jarosław Brodny, Magdalena Tutak

**Affiliations:** 1 Faculty of Organization and Management, Silesian University of Technology, 44-100 Gliwice, Poland; jaroslaw.brodny@polsl.pl; 2 Faculty of Mining, Safety Engineering and Industrial Automation, Silesian University of Technology, 44-100 Gliwice, Poland

**Keywords:** unplanned machine downtime, machine availability, mining exploitation, production efficiency, sensor-based systems

## Abstract

Underground mining belongs to immensely complex processes and depends on many natural, technical and organizational factors. The main factor that hinders this process is the environmental conditions in which it is carried out. One of the problems associated with the use of increasingly modern machines in such conditions is the issue of unplanned downtime during their operation. This paper presents the developed methodology and IT system for recording breaks in the operation of mining machines and identifies their causes. The basis of this methodology is a sensor-based information system used to register mining machinery parameters, based on which interruptions in their operation can be determined. In order to register these parameters, an industrial automation system (together with a SCADA system supervising the process) was used, which is practically independent from the operator and enables continuous registration of these parameters. In order to identify the reasons for the recorded breaks, an IT tool was developed in the form of an application in the module of the integrated mining enterprise management support system (ERP system). This application enables (with a continuously updated database) the identification of the causes in question. Thus, the developed solution enables the objective registration of machine downtime and, for most cases, the identification of causes. The acquired knowledge, so far largely undisclosed, has created opportunities to improve the utilization level of machinery exploited in the mining production process. The paper discusses the methodology, together with the IT system, for identifying the causes of machine downtime and presents an example of its application for a shearer loader, which is the basic machine of a mechanized longwall system. The results indicate great potential for the application of the developed system to improve the efficiency of machinery utilization and the whole process of mining production.

## 1. Introduction

The dynamic development of the world economy results in increased competition, which forces companies to implement more and more advanced technical and organizational solutions [1,2,3]. The aim of these activities is to gain competitive advantage and, consequently, to keep operating and developing these companies. This concerns virtually all sectors of the world economy. One of its main reasons for this increased competition is the rapid development of information technology (IT). The changes taking place in this area translate into all spheres of life and cover the entire global economy. Since 2011, these changes have been referred to as Industry 4.0—used for the first time at the Hannover Fair [4]. In addition, in recent years, concern for the environment has become increasingly important and has had a significant impact on the development of the global economy. The economy of sustainable development, combined with economic digitalization and the development of the Internet, are increasingly affecting production, trade, consumption and many other areas of life [5]. These changes are placing ever higher demands on manufacturing companies, which need to take a more rational approach to using the potential of their resources and introduce new management systems.

To meet the growing competition, companies must adapt to these changes and, above all, minimize costs and improve their efficiency [6,7]. These processes are closely related to the need for new technical and organizational solutions and effective management of resources. The effects of changes concern the whole world economy, including the mining industry. This mainly concerns the hard coal mining industry, which additionally must cope with increasing social pressure related to environmental protection and the difficult environmental conditions in which the underground exploitation of this raw material is carried out [8,9]. Due to these factors, the sector is more and more determined to implement solutions aimed at reducing production costs and making fuller use of its resources, thus improving its efficiency. A great opportunity to achieve the assumed objectives and to adapt to the trends in the global economy is the implementation, to an ever-greater extent, of IT solutions and the use of the possibilities of industrial automation systems. It should be emphasized that currently both companies and whole sectors are concerned with the introduction of digital solutions that will have a huge impact on the efficiency of both their processes and safety [10].

The transformation of enterprises towards the implementation and use of smart manufacturing systems (SMS) is based on three fundamental pillars: connectivity, virtualization and data utilization [11,12]. The digitalization of enterprises is driven by an extremely dynamic development of information technology (IT) and operational technology (OT), which are associated with the use of solutions such as sophisticated sensor-based systems, systems for recording and analyzing large sets of diagnostic data, or the use of artificial intelligence [13,14]. Sensor-based systems, which modern machines and production lines are equipped with, enable the registration of parameters of their work, which is more and more widely used to improve and optimize production processes. The analysis of these data, mostly in the form of time waveforms [15], makes it possible to identify anomalies, characteristic failure patterns and the state of normal operation of machines. The number of disturbances depends on many factors, and one of them is the conditions in which the production activity is carried out, which in the case of underground mining is of great importance. Therefore, the use of such systems, especially in the mining industry, seems to be fully justified.

Therefore, it can be concluded that the solutions used so far in other production sectors should also be increasingly implemented in the mining industry, which is perceived, at least in Poland, as conservative in terms of implementing innovative solutions. This assessment results from the tradition of underground mining, the specific working environment and many other factors that make the implementation of new solutions more complex than in other sectors. Nevertheless, changes taking place in the global economy and strong competition on the energy raw materials market make it necessary for the companies from this sector to adapt to the changes taking place if they want to continue their activities.

**Figure 1:**
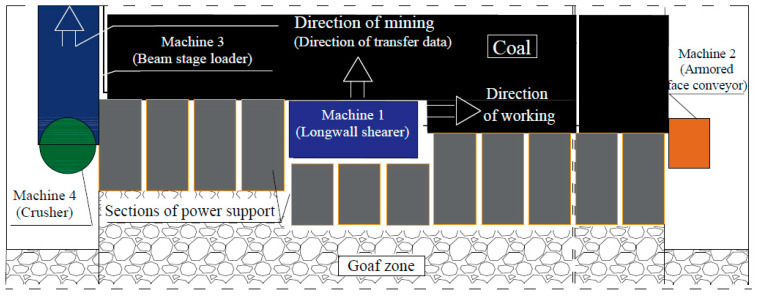
A mechanized longwall system in a longwall area.

One of the most important problems in the process of underground mining is the effective use of technical resources that are in the possession of mining companies. This mainly applies to all types of mining machinery and equipment, including those included in a mechanized longwall system. The purpose of the mechanized longwall system is highly efficient mining of the rock mass and horizontal transport of the extracted material from the face area. In addition to mine support, armored-face conveyors and beam-stage loaders and crushers, the key machine of the system is a shearer loader, i.e., a machine that directly mines the rock mass. The full and safe use of the potential of these machines is of key importance for the effectiveness of the entire mining production process. Figure 1 presents a mechanized longwall system in a longwall area.

The machines included in the system are technically advanced and adapted to exploitation in difficult underground conditions and, consequently, very expensive.

Therefore, it is in the mines′ best interest to make the best possible use of their potential, which is becoming increasingly important in the current reality. It should also be emphasized that the process of excavating the rock mass and the initial transport of the extracted material (from the face zone) are of key importance for further activities related to the operation of mines. Any disturbance of this phase of mining production has a negative impact on the entire activity of a company. The full utilization of the machinery potential of the longwall system is of crucial importance for the whole activity of the mine. Therefore, when considering the importance and timeliness of the issues related to the use of mining machinery and its impact on the efficiency of the entire process of mining production, it is reasonable to take action to develop a method for analyzing the degree of utilization of these machines. Their availability was adopted as a measure, i.e., the ratio of their actual working time to the normative time. In this respect, it is crucial to determine the number of unplanned breaks in the working time of these machines and their causes.

The article presents the developed methodology for testing the availability of mining machines, based on diagnostic data recorded by the industrial automation system, using state-of-the-art sensor-based systems. Based on the analysis of these data, it is possible to determine the availability of these machines and the time structure of the recorded breaks in their operation. Understanding the causes of interruptions in the work of mining machines is of key importance for the whole process of improving and optimizing their operation. In addition, mining machines usually work in a four-shift system, where one shift is the so-called maintenance shift. Therefore, it is expected that these machines are used as much as possible during working shifts.

In the next stage, an IT tool was developed to identify the causes of unplanned breaks. This tool—in the form of an additional application constituting a part of the Module of Production Means Management, contained in the Production and Technical Complex (PTC)—was integrated with the SZYK 2 (ERP) system used in mines. The databases on used machines, their components and their possible failures, implemented in this module, considerably simplified and facilitated the process of identifying reasons for downtime in their operation.

The article presents the research methodology and discusses the developed IT tool for analyzing the causes of downtime in the operation of mining machines. It also presents an example of its direct application in one of Polish hard coal mines to study the working time and identify the causes of breaks in this working time for a shearer loader. The research covered one month of operation of the machine in question (74 working shifts).

Therefore, the main objective of the presented research was to develop and implement a method for reliable identification of breaks in the operation of mining machines and of their causes. However, IT tools and an industrial automation system were used to obtain the most objective results of the developed method.

The results indicate that the shearer loader′s capabilities were not used satisfactorily, which resulted from a number of unscheduled breaks in its operation. The industrial automation system was used to analyze the structure of these breaks and to specify the availability of the shearer leader during working shifts. The causes of interruptions in the shearer′s work were assessed using the developed tool. The process of registration of downtime causes in the shearer′s work was carried out in a semi-automatic system, in which solutions used in the developed application supported the work of dispatchers, who entered information about unscheduled breaks.

The summary of breaks recorded in the application was compared with the results obtained from the industrial automation system, which made it possible to assess the effectiveness of the developed tool. The results obtained for the examined period were found to be optimistic and satisfy the users. The developed tool made it possible to gain knowledge, hitherto largely implicit, concerning the causes of interruptions in the shearer loader′s work. In addition, the causes of these breaks were assigned to four groups (mining, mechanical, electrical and hydraulic) adopted in the research, which should facilitate further analyses in order to eliminate and/or reduce them. The adoption of such groups of causes was due to the fact that mining companies have groups of specialists dealing with these issues.

## 2. Literature Review

The problem of applying innovative solutions in the mining sector is increasingly being taken up by researchers and thus also presented in publications. More and more publications also concern the effectiveness of machinery utilization and the analysis of unplanned downtime, which have a significant impact on this effectiveness. It should also be noted that the problem of limiting unplanned breaks and identifying their causes concerns practically all economic sectors, where machines are used. For this reason, the presented literature review will include only the most significant items related to determining the effectiveness of machine utilization and the study of unplanned downtime, in the scope of which the number of published studies is much smaller.

The problem of downtime in the production process, which has an impact on the effectiveness of machinery utilization, is an important part of an extensive issue related to the Total Productive Maintenance (TPM) strategy and the Overall Equipment Effectiveness (OEE) model. Downtime, including unscheduled breaks, is an important topic in manufacturing because of its relationship to the efficiency and profitability of the implemented processes. Reducing downtime in these processes, which also applies to hard coal production, is a necessity as it serves their more effective implementation [16].

According to Hechtman, downtime itself can be defined as any event that results in stopping planned production for a certain, indefinite period of time [17]. Machine downtime during a given process can be divided into planned (maintenance, changeover and reconfiguration) and unplanned (e.g., failures). Each machine downtime, either planned or unplanned, is intuitively costly for an organization and often difficult to estimate [18].

The available literature presents the results of very few studies related to machine downtime that affect manufacturing operations and its efficiency. A vast majority of these publications are devoted to the OEE model for various industries [19,20,21,22,23,24,25,26], including the mining industry [24,25,26].

The issue of machine downtime is discussed in a publication [27] that presents the results of an analysis on drilling machine downtime. The analysis was conducted to determine which components and what types of problems (maintenance and/or reliability) contribute to downtime. The findings made it possible to identify strategies, maintenance and/or reliability designs that should be used to reduce machine downtime (in this case, a drill). Fitchett [28] pointed out that one way to monitor machine downtime is the overall performance of a single piece of equipment, governed by the combined impact of three OEE factors: availability, performance index and quality. Fischet, on the other hand, concluded that this single element affects the performance of the entire enterprise. Similar conclusions were drawn by Fujishima et al. [29], who found that a key to quickly delivering orders to customers is rapid maintenance of the production line during unplanned interruptions, which leads to improvements in the production process. This issue has been solved quite clearly in companies where the maintenance problem is implemented in one of the so-called maintenance shifts. A similar approach was presented in another work [30], where the authors conducted analyses on the impact of a preventive procedure on the reduction of downtime in the case of the Multi-Leaf Collimators machine (MLC).

The trend of maintenance change was also presented by Mohan et al. [31]. The authors developed an intelligent machine-learning-based approach for comprehensive productive maintenance to achieve zero downtime in industrial machines. The proposed system provides promising results that increase the average time between failures due to contaminated oil by 800%. The proposed maintenance approach has been implemented in a high-pressure hydraulic sand molding machine in an automotive foundry producing gray cast parts. By contrast, Fadeyi et al. [32] used a reliability model to solve the problem of downtime in a hygiene products manufacturing company. They identified subsystems of the production system responsible for downtime and then applied the reliability model to reduce downtime. Prombanpong et al. [33] presented a numerical approach to determine an appropriate number of buffer stocks to mitigate the adverse effects of downtime in an automated transmission line on its efficiency. Nwaya et al. [16] conducted a study on scheduled machine downtime in a plastic factory in Nigeria. The study revealed that pit stops account for 25.64% of the planned downtime. The study also showed that it is possible to estimate downtime and thus take action to reduce it.

The literature review proves that the problem of machine downtime is extremely important for the effectiveness of the production process, but it is rarely discussed in the literature. So far, no research has been carried out on the causes or duration of unplanned machine downtime in hard coal producing enterprises (mines). Additionally, unplanned machine breaks, as Sivaselvam and Gajendran [34] prove, significantly affect the efficiency and planned execution of the production process.

Current research on the effectiveness of the mining machinery utilization indicates large reserves in this respect [35,36,37,38,39,40,41,42,43]. Quite a few works deal with open-pit mining, presenting analyses of the efficiency of loaders, excavators and crushers. On the other hand, there are very few studies on the efficiency of the use of machinery in underground mining. One example is a study [40,42,43] that presents the results of efficiency evaluation (OEE model) of a longwall shearer from the Parvade coal mine (Iran). The problem of determining the efficiency of mining machines operating in coal and bauxite mines was also referred to in another work [44,45,46].

To sum up, the problem of full machinery utilization in production processes is important for their efficiency. On the other hand, unplanned interruptions pose a huge threat to the efficiency of these processes. Therefore, the problem of identifying them and determining their causes is a key element in the process of improving the degree of their utilization. It is also crucial for underground hard coal mining, for which there are no studies dedicated to this subject. Therefore, it is fully justified to discuss the subject of identifying unplanned downtime in the operation of mining machines and their causes. The application of the developed IT solution and the verification of its operation on the basis of diagnostic parameters of these machines recorded by the industrial automation system is a novel and original approach to this subject.

## 3. Materials and Methods

The chapter presents brief characteristics of the industrial automation system and computer tool used to analyze mining machine downtime.

### 3.1. Characteristics of the System for Identifying Machine Downtime and Its Causes

The conducted research, the results of which are presented in this paper, was based on the use of industrial automation systems and sensor-based systems (which directly register studied physical quantities) to analyze the operating parameters of mining machines. Based on the analysis of these parameters, it was possible to determine the actual working time of these machines. By referring to the normative working time, it was possible to determine breaks in the operation of these machines and their availability.

The very concept of availability, and productivity and quality of production, is related to the TPM strategy. All three values are used as basic factors in determining the value of OEE index, which is a measure of the effectiveness of TPM strategy implementation [47,48].

In the presented work, the first of these factors—availability—was adopted. In order to determine it as objectively as possible, sensor-based systems were installed in the studied machines to register their parameters. This registration is continuous and independent from the human factor, which means that the data can be used to analyze the working time of a given machine. It is crucial in the case of underground mining because these difficult and, oftentimes, unpredictable environmental conditions can adversely affect workers and technical equipment, causing many unplanned breaks in the working time of machines.

When the registration of breaks and the identification of causes were done by dispatchers without using data from the industrial automation system, large quantitative discrepancies were reported, which made it difficult to identify causes.

Therefore, the results determined on the basis of the recorded operating parameters were the inspiration to develop a system for identifying the causes of these interruptions.

**Figure 2:**
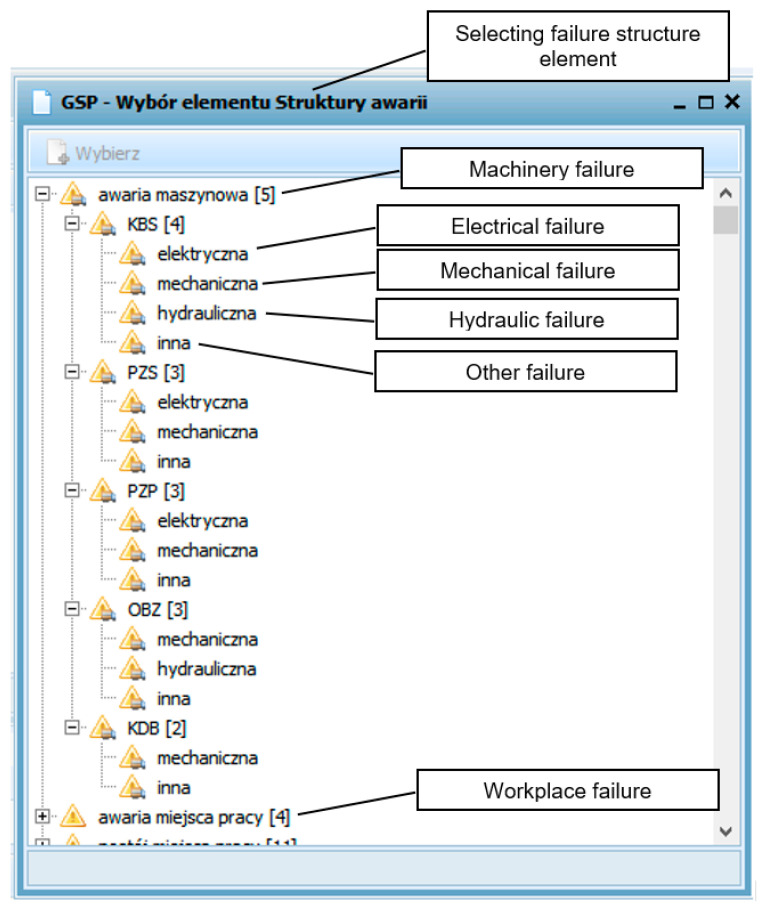
View of the script for selecting failure structure elements (unscheduled downtime).

To reliably identify the causes of breaks during machinery operation, an integrated ERP class system (of SZYK2 type) used in mines was employed. An application developed to identify the causes of unplanned machine downtime was added to the Production Means Management Module, contained in the Production and Technical Complex (PTC) of the SZYK2 system. This application enables dispatchers to precisely identify causes of breaks in the longwall system’s machines. The view of the script for selecting the elements of failure structure is shown in Figure 2.

The very process of registration of unplanned downtime in the operation of machines has a semi-automatic character. When a specific parameter (or group of parameters) characterizing the operating state of the machine reaches a certain value, the system signals such a state to a dispatcher. In the case of the shearer loader, such parameters were the speed of the shearer and the currents consumed by the shearer head engines. If the speed was “zero” and the currents did not exceed the values for idling, we were dealing with an unscheduled break in the operation of this machine. For other machines, these criteria are appropriately matched to them and can be changed as needed. The occurrence of such an event makes a dispatcher fill out a card, shown in Figure 2, to record the cause of this interruption. Basic information about causes can be also extended by a short comment by a dispatcher. This is especially advisable in case of longer breaks, when a dispatcher has more time to determine and/or comment on the cause of a given break. The communication panel contains a database of machines, their components and parts, and the most frequent causes of breaks, which makes a dispatcher’s work much easier and faster. The application also has the possibility to constantly expand this database, with new events not yet identified.

**Figure 3:**
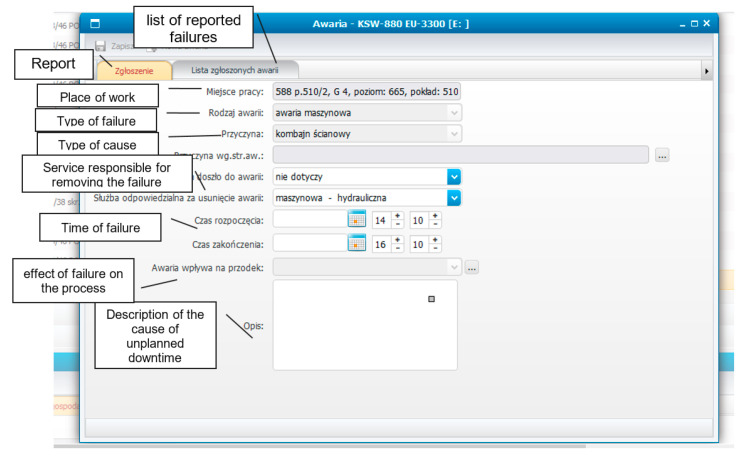
The view of an IT tool card for recording unplanned machine downtime.

In this application, each dispatcher logs in, using an individual and dedicated identifier to preserve, to some extent, his anonymity. This limits the possibility that outsiders will obtain information about a dispatcher. This approach creates the possibility of acquiring knowledge, very often secret, of the real reasons for machine downtime. Thus, it provides the possibility to react to the disturbances in machine operation. The page view of the IT tool for recording unplanned machine downtime is shown in Figure 3 (original view with description in Polish).

An important element of this card is the ability for a dispatcher to record additional information (in the description field). This information requires additional analysis, but it is important for gaining additional knowledge of the real causes of interruptions or events that have occurred. As mentioned earlier, the process of determining the causes of interruptions in the operation of mining machines was a consequence of their objective quantitative evaluation by the industrial automation system. Based on these results, quantitative verification of these interruptions with the reasons given by the dispatchers was also carried out.

The results obtained for the shearer loader in one of the Polish coal mines (for a period of one month) (presented in Section 4) showed that the biggest problem occurred in the case of short breaks in the shearer’s work (up to 30 s). However, for longer breaks, the system was able to specify the reasons for most of the identified breaks.

### 3.2. Characteristics of the Process of Machine Operation Data Acquisition and Analysis

**Figure 4:**
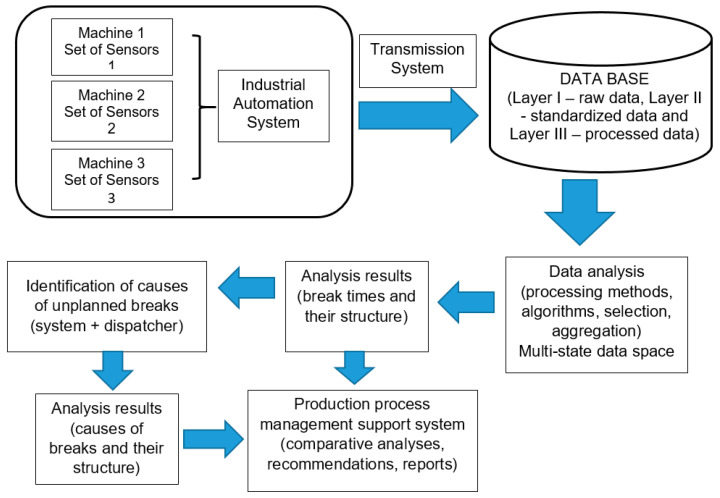
Diagram of the process of acquiring and using data on the parameters of machine operation to support their work management.

A very important element of the methodology for determining the causes of interruptions, especially in terms of verifying the effectiveness of this identification, was a system for obtaining and analyzing the operating parameters of the machines under study. Figure 4 shows a diagram of the process of obtaining data on the operating parameters of these machines. It constitutes one of the basic parts of the whole information system to identify breaks and their causes.

It shows the way of building databases for individual data layers. Of particular importance in this process is the removal of gaps in the acquired data and the qualification of data for further processing. The result of this activity is feeding this part of the data warehouse in which the processed database is located. These data are the basis for further analytical processing, which helps determine breaks in the operation of these machines and, for example, their load, as well as other characteristics. It should be remembered, however, that the primary importance in this whole process is attributed to the system of sensors, installed in the studied machines to acquire data on the parameters of their operation.

The basic component of the developed system is a data warehouse (HD), which includes a number of relational databases. They contain input data about the parameters of tested machines, calculation algorithms and result databases. The input data are particularly important as the quality of results depends on their quality and later on the accuracy of an analytical process. In order to eliminate the problems connected with data acquisition and to provide system processing mechanisms, the following data layers were introduced for subsequent transformations (in the database):

- Raw source data layer (off-line data, file databases and on-line data), standardized source data layer (HD, layer I),
- Selected, standardized source data layer (HD, layer II),
- Processed data layer based on the interval model necessary to determine the OEE indicator (HD—layer III).

The OpenEye system was used to manage the entire IT system, especially the acquired data. This tool uses elements of the SCADA system supervising the course of the technological process. It is built with a number of tools enabling quick data analysis and visualization of results. The OpenEye system contains built-in visualization means and cooperates with any relational databases such as Oracle, MS SQL and PostgreSQL.

The final effect of using the developed systems is the ability to identify unplanned downtime and its causes. An important advantage of this tool is also the fact that it provides an opportunity to exchange views and comments of all persons involved in the process of mining exploitation. This applies both to employees and dispatchers as well as capital group management. Additionally, access to data and analysis results can be regulated in any manner.

## 4. Results

To determine unplanned breaks in the operation of mining machines and, at a later stage, identify their causes, the authors used the time waveforms of diagnostic signals recorded by the industrial automation system, supported by a set of specialized sensors. The analysis of these data also helped to determine the availability of a given machine and other parameters of its operation.

In the case of a shearer loader, the signals are usually the time waveforms of the feed speed and the currents consumed by the engines of a shearer loader and, as an additional parameter, the position of this shearer loader in the longwall, defined in relation to the powered roof support section. Depending on the efficiency of the industrial automation system and the transmission network, any of these signals may be used for analysis.

**Figure 5:**
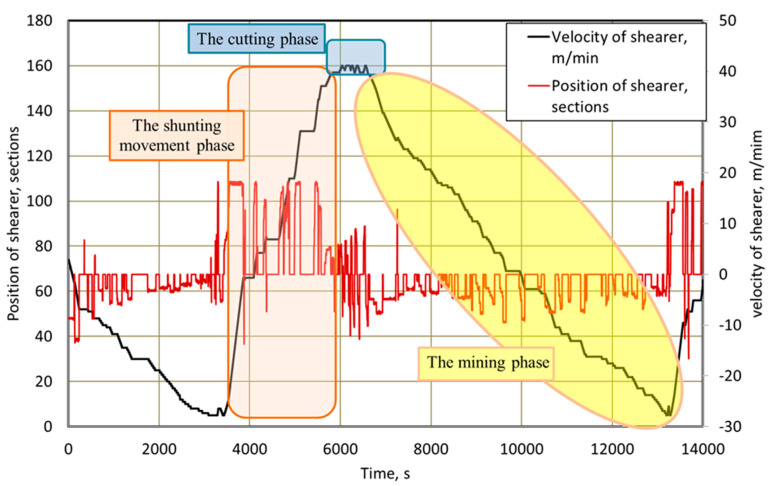
Time waveforms of changes in the position and speed of the shearer loader in the longwall face.

In general, for the analysis of shearer loader operation, the most frequently used parameters are the time waveforms of changes in its position in the longwall face and in the rate of feed speed (Figure 5). The presented waveforms comprise one working cycle, including three phases: shunting, cutting and mining. The “plus–minus” signs at the feed speed denote the directions of the shearer loader’s movement.

**Figure 6:**
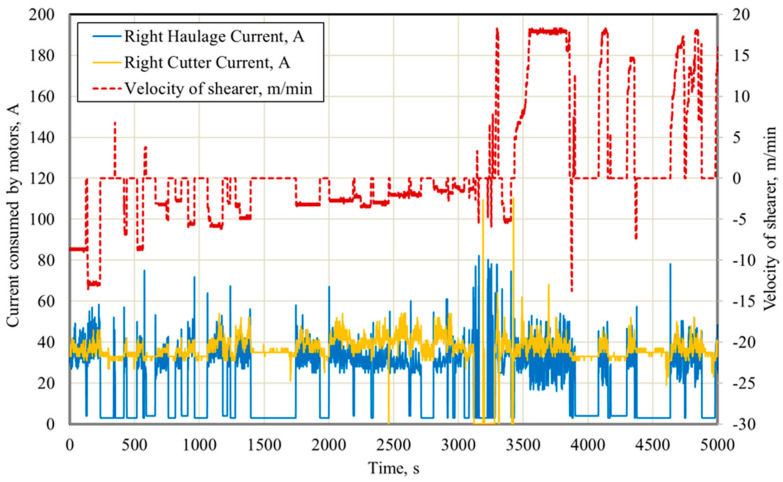
Time waveforms of the shearer loader’s speed and the intensities of currents consumed by the right motors of the shearer’s arm and feed.

**Figure 7:**
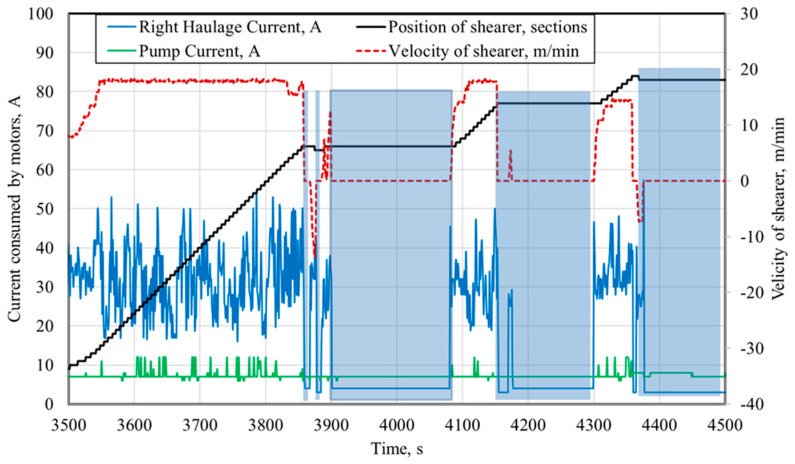
Time waveforms of the shearer’s speed and position in the longwall face and of the intensities of currents consumed by the right motors of the feed system and the hydraulic pump.

**Figure 8:**
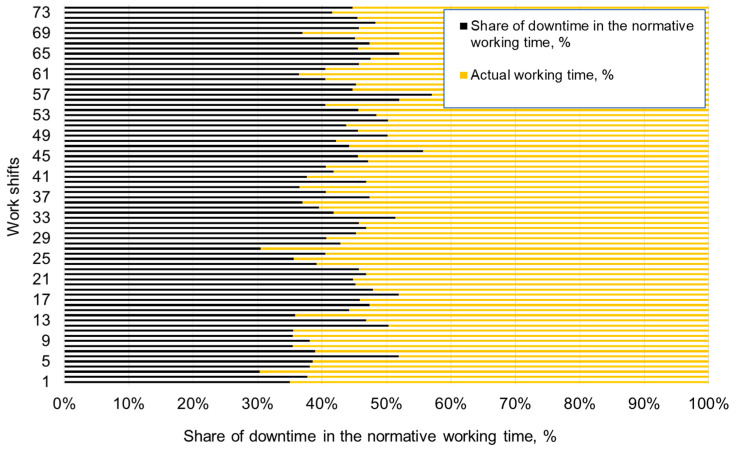
Percentage comparison of time of unscheduled downtime in the shearer’s work during individual shifts.

The industrial automation system used in mining machines also enables the registration of many other parameters of the shearer loader’s work, which may be used for a more complex analysis of its operation. Figure 6 presents the time waveforms of the shearer loader’s speed and the intensities of the currents consumed by the right motors of the shearer loader’s arm and feed, for the initial phase of its operation (83 min and 20 s) presented in Figure 7. Figure 8 presents the time waveforms of the shearer loader’s speed and its position in the longwall face, as well as the intensities of the currents consumed by the right motors of the shearer loader’s feed and the hydraulic pump. They represent a part (75 min) of the shunting movement phase, during which the shearer loader is moving to the beginning of the working face, without excavating the rock mass. Selected unscheduled breaks in the shearer loader operation during this time are also marked in this figure.

When analyzing the time waveforms presented in Figure 7, it can be stated that for this part of the shearer’s work, three longer breaks (180 s + 144 s + 124 s) and two shorter breaks (2 × 9 s) were recorded, and the total time of marked breaks was 466 s.

The time waveforms of the shearer loader’s work parameters recorded by the industrial automation system were the basis for determining the time structure of unplanned interruptions in its operation. On the other hand, the application of the developed system for identification of the causes of these breaks enabled a general analysis of the structure of these causes (mechanical, electrical, mining and hydraulic). In many cases, thanks to a more detailed description, it also enabled more detailed identification of causes.

As mentioned earlier, the research was carried out for one month of operation of the shearer loader in the longwall of one of the underground coal mines. Thus, the total time of the research encompassed 74 working shifts, i.e., 444 working hours (normative time). A percentage comparison of the duration of unscheduled breaks in the shearer’s work during particular working shifts in the analyzed period is presented in Figure 8 (share of downtime in the normative working time and the actual working time).

Based on the results, the percentages of unscheduled downtime of the shearer loader during individual shifts were found to be different. In general, these values ranged from 30.29% (for the third shift) to 56.99% (for shift 57). The average percentage of unscheduled breaks during the study period was 43.47%, which means that, on average, instead of 21,600 s, the shearer loader worked for 9389.6 s per working shift. Therefore, it can be assumed that the potential and possibilities of the shearer were used in this longwall only slightly more than 56% of the time.

**Figure 9:**
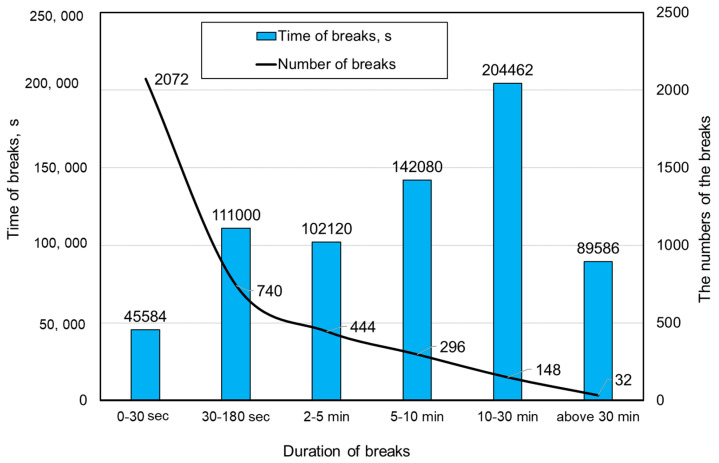
Summary of the time quantitative structure of unscheduled breaks in the shearer loader operation.

The use of the industrial automation system also made it possible to determine the time structure of unplanned interruptions in the shearer’s work. In this analysis, the interruptions were divided into six time categories, and the results are presented in Figure 9, taking into account the number of breaks registered by the system as well.

The results clearly indicate that short breaks (up to 180 s) were most frequently recorded, which jointly constituted 75.35% of all breaks, while as far as time is concerned, the most significant were breaks between 10 and 30 min, accounting for 20.45% of the total time of the registered breaks.

Statistical data on the number and temporal structure of breaks provided the basis for assessing the effectiveness of the system for identifying the causes of these breaks. Breaks lasting less than 30 s (the so-called micro-breaks) were excluded from the identification process. This was due to their short duration and large number of interruptions, which made the process of identifying their causes much more difficult. These breaks constituted 6.5% of the total break time and were omitted from the presented analysis.

**Figure 10:**
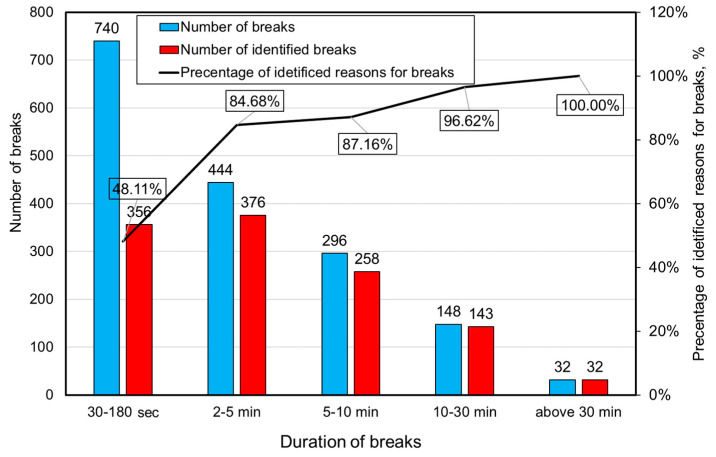
Summary of the number of identified breaks in the shearer’s work for particular time categories.

In the next stage, the application developed to identify the causes of unplanned breaks in the shearer loader work was utilized. The summary of the number of identified interruptions in the shearer operation for particular time categories for the examined period is presented in Figure 10.

**Figure 11:**
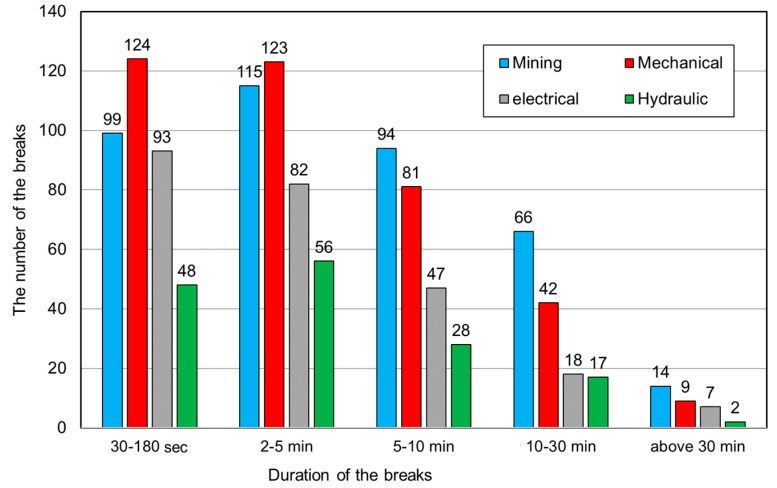
Types of causes of breaks in the shearer loader’s work for particular time categories.

**Figure 12:**
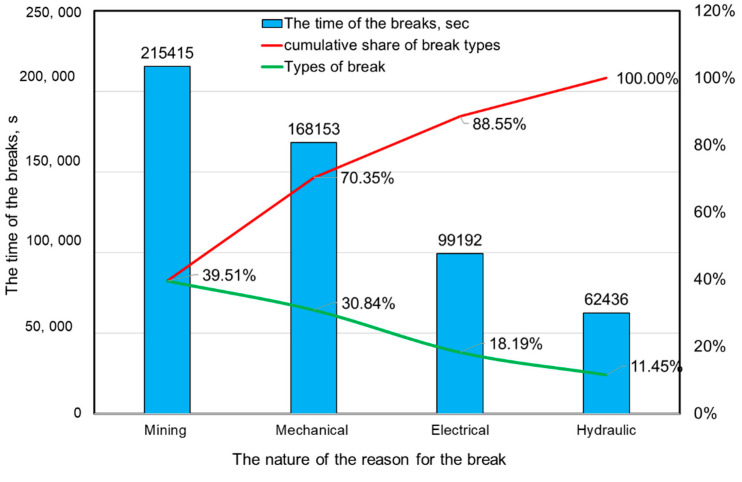
Time of individual types of break causes with their percentage share (cumulative share of break types).

Based on the results, it may be stated that the developed and applied system for identifying the causes of interruptions in the operation of mining machines in the case of the shearer loader works very well in the scope of longer breaks (above 2 min). In the case of their duration above 30 min, the system enabled the identification of all of them. For shorter durations, the effectiveness of the system was slightly lower, but the results obtained can be considered satisfactory. In the case of breaks of up to 180 s, the effectiveness of identification was 48.11%, which, considering the short duration of those interruptions, is not a bad result. The system for identification of break causes also made it possible to assign them to one of four groups (mining, mechanical, electrical and hydraulic). Assigning a given break to an appropriate group was done by the dispatcher (by indicating in the application the nature of the break cause). In the vast majority of cases, the dispatchers explicitly defined the reasons for the breaks in the developed application. In cases where there was no such assignment, the assessment was carried out based on the description of the event causing a given interruption. The results of this analysis are presented in Figure 11, while Figure 12 presents a time compilation of break types with their percentage share.

The results show that among the identified causes of the recorded breaks, mining causes accounted for the largest number. They accounted for 39.51% of the total time of the identified break causes. In turn, 30.84% of the causes were mechanical in nature and the least (11.45%) were hydraulic (Figure 12). When considering the time structure, mechanical causes dominated for shorter breaks (30 s–5 min), while mining causes dominated for longer breaks (Figure 11).

It should also be stressed that these causes have their source mainly in complicated and dangerous mining and geological conditions, under which exploitation is carried out. In most cases, their cause was not related to machine failure since shearers used nowadays are at a very high technical level, and their high reliability and efficiency ensure failure-free operation at high intensity of exploitation. However, environmental conditions, the human factor and organizational problems lead to various types of events, resulting in unplanned breaks in the operation of these machines.

Therefore, it can be concluded that the application of the developed IT tool made it possible to determine causes for more than 70.18% of unplanned breaks in the shearer loader’s work (duration above 30 s). When referring to the duration of these interruptions, this result amounts to 78.46% of the total time of breaks registered by the industrial automation system.

## 5. Discussion

Issues related to the effective use of machinery in production processes are being faced by many industries. Reducing all types of breaks, especially unplanned downtime, is one of the main tasks of maintenance departments. In the case of underground mining, this issue is more complex due to difficult conditions in which these processes are carried out. However, also in this case, mainly due to economic reasons, it becomes necessary to strive for the best use of the potential of machinery owned by a company. Nowadays, these processes are supported by IT solutions, to an ever-greater extent, together with modern sensor-based systems, being the basis for industrial automation systems.

The developed and implemented methodology for identifying the causes of breaks in mining machines is a positive example of using the industrial automation system (based on sensor systems) and IT solutions to solve a current and important problem.

The main advantage of this methodology is the use of an objective source of information on actual breaks in the operation of machines, namely, diagnostic data from their operation. This approach guarantees independence from, very often, subjective evaluations of dispatchers or other workers. The issue of criteria selection, on the basis of which the state of unplanned breaks will be determined, should be specified depending on the specificity and nature of machinery operation. However, relying on diagnostic data, continuous and automatic registration provides great opportunities to determine these criteria in accordance with the expectations and requirements of the managers of a given process.

The developed IT solution in the form of an application, which is already functioning and used in the mining industry integrated management system in the enterprise (ERP class), makes the process of its implementation quite easy and not as expensive. Additionally, the interface of this application is adapted to the already functioning IT solutions in the mining industry. Therefore, both the economic aspect associated with the implementation of the developed solution and its operation should not constitute a significant barrier in the process of its wider use.

However, as was mentioned before, the human factor may be a problem as the mining sector is rather distrustful towards new solutions. In addition, as was shown, the issue of objective registration of the quantity and times of unplanned breaks of machines and identification of their causes shows that it may constitute a considerable problem for the management of mines and for the maintenance of these machines.

However, the developed methodology and information system enabled the acquisition of new knowledge (including classified knowledge) in the field of:

- Obtaining reliable quantitative data on breaks in machine operation (their distribution and time structure),
- Identifying causes of most breaks during machine operation and recorded by industrial automation systems,
- Using the above information to develop an effective system supporting the process of effective management of technical resources in a mine (e.g., by identifying types of break causes).

The findings provided a lot of new information on the operation of the shearer loader and thus broadened the technical and organizational knowledge in this respect. Information about a large number of registered and unplanned interruptions in its operation, as well as the issue of short breaks and the problem with identifying their causes, is important in this case. In this respect, the developed tool made it possible to identify the causes of these breaks to a very limited extent. According to the authors, this problem can be explained, and it requires greater involvement on the part of the operators of these machines and further acquisition of classified knowledge regarding the operation of mining machines.

It is also worth emphasizing that the effectiveness of the developed methodology and implemented application is based on the analysis of large sets of diagnostic data. Moreover, the developed application used databases that can be continuously updated with regard to the machines used in the process; their construction, parameters, possible causes of failures and various types of interruptions; and other information. The versatility of the developed application means that it can be easily extended to include other machines involved in the mining production process, and supplemented with necessary data and information.

In conclusion, the developed methodology, together with the IT tool, made it possible to solve an important problem related to the operation of machinery in a mining company. The knowledge has been expanded in both cognitive and utilitarian areas, as well as in terms of improving the efficiency of machinery utilization during underground mining production. Thus, the information obtained, and the knowledge derived from it, should become valuable for both the management and employees of mines and for machinery manufacturers. The study showed problems connected with the exploitation of mining machines, and for some of them, the reasons for these problems, which should facilitate their solution.

## 6. Conclusions

The paper presents the results of research on unplanned downtime in the work of mining machines. The analysis was based on methodology developed using an operator-independent system for recording diagnostic parameters in the operation of these machines and a computer system for analyzing the obtained data. The main result of these activities is the identification of unplanned interruptions in the operation of the analyzed machines, and the determination of their time structure. Based on this information, an IT tool was developed to identify the causes of these breaks. This process is carried out in cooperation with a dispatcher who, based on recorded breaks, contacts machine operators or employees directly supervising the exploitation process (at the longwall) and, using the developed interface, identifies the causes of these breaks.

The developed methodology and IT system were used in one of the underground coal mines to analyze the operation of the longwall shearer. The research conducted and the results obtained allowed the authors to draw the following conclusions:

- The developed system enables temporal identification of all unplanned interruptions in the operation of tested machines. Its accuracy depends only on the frequency of data recording, which in the presented example is 1 Hz and is sufficient for the realization of the research objective.
- As far as the causes of recorded breaks is concerned, the efficiency of the system also depends on the work of a dispatcher. As per the presented example, positive results were obtained in terms of identifying the causes of longer interruptions. For downtime lasting less than 180 s, the identification efficiency was found to be lower. This is due to their short duration and larger number of interruptions, which limits a dispatcher’s ability to identify them. However, the influence of these (short) breaks on the availability of the shearer was small (in terms of their aggregate duration).
- The obtained results showed low availability of the shearer under study. On many of the studied shifts, the shearer did not exceed even 50% of its normative working time, which is an unfavorable result.
- The analysis of the structure of causes of registered breaks (in the identified part) makes it possible to state that they were most frequently caused by mining and mechanical problems. This particularly concerns longer breaks, which should be taken into account in the process of managing services responsible for this area of exploitation.
- The developed method and IT tool can be applied to a much larger number of machines (practically all machines used in a production line).

The methodology and the tool presented in the article are an example of applying modern IT solutions in the mining industry in order to improve the efficiency of production processes. For the mining industry, this solution represents a new approach to the identification of unplanned machine downtime and their causes and should be widely implemented in mines. It should be remembered, however, that this solution, in conjunction with the system for recording the working time of machines (their operating parameters), allows for a very objective assessment of this downtime, which may not be fully accepted by employees. On the other hand, at the current stage of implementation, the identification of the causes of downtime is also dependent on the work of a dispatcher.

## Data Availability

Not applicable.
